# Outsmarting Olfaction: The Next Generation of Mosquito Repellents

**DOI:** 10.1289/ehp.113-a468

**Published:** 2005-07

**Authors:** Charles W. Schmidt

The world’s most dangerous animal weighs about two milligrams and pursues its human prey at speeds of barely a mile per hour. Surprised? Don’t be. The dubious honor belongs to the lowly mosquito—a fragile creature whose bite infects millions with lethal diseases, such as malaria, dengue, and West Nile encephalitis. For centuries, humans have slathered on insect repellents to deter the buzzing menace—the first recorded repellents were documented by Herodotus around 400 B.C. But these products have always been far from optimal. Even DEET (*N,N′*-diethyl-*n*-toluamide), the world’s most popular and efficacious repellent, has numerous shortcomings: it can require frequent applications, it must be applied to all exposed body parts, and it won’t protect against some dangerous mosquito species, including *Anopheles albimanus*, the chief malaria vector in Central America.

Today, the need for more effective repellents is increasingly urgent, experts say. According to the World Health Organization, global climate change is expanding mosquitoes’ range, heightening the risk of disease for millions of additional people. The Centers for Disease Control and Prevention notes that dengue and West Nile virus are both moving from developing countries towards the United States, where concerns over mosquito exposure are rising. Malaria—which by various estimates kills between 1 million and 3 million people worldwide each year—is also a growing problem in many regions. This is in part because *Plasmodium*, the mosquito-borne parasite that causes malaria, is fast becoming resistant to existing treatments, such as chloroquine.

Many experts believe that better repellents could help to control mosquito-borne diseases. These next-generation repellents must be effective against anopheline mosquitoes that carry malaria. They should also be cheap and nontoxic, and should last long enough to protect humans as they sleep, when they are most vulnerable.

Where will these repellents come from? The answers, scientists increasingly say, will be found in genomic research. According to this view, knowledge of the genes and proteins that mosquitoes rely on to sense their environment could lead to new repellents that directly interfere with the insects’ ability to detect human beings.

## A Sense of the Future

Today, genomic information about mosquitoes is accruing rapidly. Scientists with the International *Anopheles* Genome Project, a consortium based at the Pasteur Institute in Paris, have already decoded the genome of *An. gambiae*, the dominant malaria vector in Africa. Another genome, for *Aedes aegypti*, a vector for both yellow fever and dengue, is now being sequenced with funding from the National Institute of Allergy and Infectious Diseases. Using these data sets, scientists are identifying the genes that control mosquito sensory systems, including the olfactory system.

Mosquitoes rely on smell to guide them towards mates, food, and of course, sources of blood meals. The process is highly specific. For example, only female mosquitoes are attracted to blood sources, which they require as nourishment for their eggs. (Mosquitoes’ main nutriment comes from other sources, mainly plant nectar.) Olfaction is also species-specific. *An. gambiae*, for instance, prefers to bite humans. “You could be in a room full of cows, and that mosquito will find you and bite you,” says Laurence Zwiebel, an associate professor of biology at Vanderbilt University. Because this species has evolved to target humans, it will stay indoors where it can get to them more easily, he adds.

To better understand the molecular biology of mosquito olfaction, consider the following scenario: You’re asleep in the summertime, in a warm room with an open window. Your body and the bacteria that reside on your skin are giving off a molecular cloud of human-specific odorants. These molecules drift through the air and soon reach a mosquito perched on the wall. In an instant, odorant-binding proteins (OBPs) located on the creature’s antennae bind the molecules and transport them towards receptors located on the surfaces of the olfactory neurons. The odorant–receptor linkage activates a cue in the mosquito’s nervous system, which alerts the insect to your presence. Moments later, the mosquito lands on your unprotected shoulder and begins to feed.

OBPs are now widely viewed as one of the most likely targets for next-generation repellents. Compounds that interfere with these proteins could block mosquitoes’ ability to smell and thus detect humans. “This really is a unique approach,” says Leslie Vosshall, head of the Laboratory of Neurogenetics and Behavior at Rockefeller University. “Since we now know quite a bit about the basic workings of insect olfactory systems, we can focus our search for new repellents that interrupt proteins known to be important for smell. We’re optimistic that this rational approach to repellent design will uncover new compounds that are safer and more effective than those that are currently available.”

## Targeting Olfactory Proteins

Vosshall has found that the function of a wide range of odorant receptors in insects depends on a single type of protein “coreceptor” that facilitates the binding of the OBP–odorant complex to the receptor. Working with fruit flies, she has shown that mutations in this protein, which in fruit flies is called Or83b, knocks out the insect’s sense of smell altogether. The protein, she says, is highly conserved across many different species; in mosquitoes, it now goes by the name GPRor7 (until recently, it was known as AgOR7). In recent studies, Vosshall generated a strain of fruit flies that lacked the *Or83b* gene and hence the ability to smell. This finding, remarkable in its own right, was rendered even more so by a subsequent discovery: when she replaced the missing *Or83b* gene with the *GPRor7* gene obtained from mosquitoes, the fruit flies’ olfactory system was restored to a normal state. This means GPRor7 substituted for Or83b, even though mosquitoes and fruit flies diverged more than 250 million years ago.

Based on these findings, Vosshall suggests that GPRor7 is a plausible target for a mosquito repellent. Knock out this protein, she says, and a mosquito will be unable to smell anything—humans included. Her findings are published in the 22 February 2005 issue of *Current Biology*.

Zwiebel and his colleagues are taking a different tack. Instead of targeting a single protein that regulates mosquitoes’ entire sense of smell, he’s homing in on the olfactory proteins that bind human odorants only. In the 15 January 2004 issue of *Nature*, Zwiebel published results showing that one olfactory receptor in *An. gambiae*—a protein known as Or1—preferentially binds 4-methylphenol, one of roughly 300 compounds found in human sweat. Moreover, the Or1 receptor was found only in female mosquitoes—further evidence of the intricate specificity in the insect’s olfactory machinery.

Today, Zwiebel and his colleagues are striving to identify other human-specific odorants with the goal of creating repellents that inactivate groups of these targets simultaneously. This approach lessens the likelihood that mosquitoes will develop resistance to the repellents, he says. “It’s almost impossible for resistance mutations to appear in four to five receptors concurrently, and they would not make their way into the mosquito population,” he explains. “What we really want is a toolbox of behaviorally disruptive olfactory compounds,” he adds. “This would allow us to fine-tune repellent blends for specific geographic regions, where mosquito populations and olfactory mechanisms might vary.”

The future repellents mark an additional departure because they could be incorporated into time-release systems that put active ingredients into the air. Thus, there would be no need to apply the repellents to the body; instead, they would protect a living space in its entirety. This is important for those mosquitoes—*An. gambiae* in particular—that prefer indoor environments and sleeping humans.

## Scratching the Surface

Experts agree that no new repellent will necessarily be a panacea for mosquito control. Andrew Spielman, a professor of tropical public health at the Harvard University School of Public Health and author of the book *Mosquito: A Natural History of Our Most Persistent and Deadly Foe*, suggests that all repellents pose a fundamental dilemma: mosquitoes deterred from one protected person will simply flock in greater numbers to another who is unprotected. “What drives the force of disease transmission is the biting rate,” he explains. “If half the people are being bitten by all the mosquitoes, then those people and any mosquitoes that feed on them will wind up being terrifically infected.” But Spielman acknowledges that repellents can be very useful—“I’m just not sure about who specifically is going to benefit from them,” he says.

When posed with this question, Zwiebel responds that health officials must take steps to ensure that no one is left unprotected. “That’s a challenge we need to accept,” he says. “We need to make sure these products are universally available and economically affordable.”

In the end, new repellents will be just part of a broader strategy to control mosquitoes, a strategy that Spielman says must also incorporate better environmental management, housing improvements, and greater use of insecticide-treated bed nets. Meanwhile, it’s hard to say which type of rationally designed repellent is likely to emerge first. Will it target one protein, as Vosshall suggests it could, or will it target several, as Zwiebel says it must? “At the end of the day,” Zwiebel says, “everyone is going forward with the best of intentions, and we just have to see what comes out.”

## Figures and Tables

**Figure:**
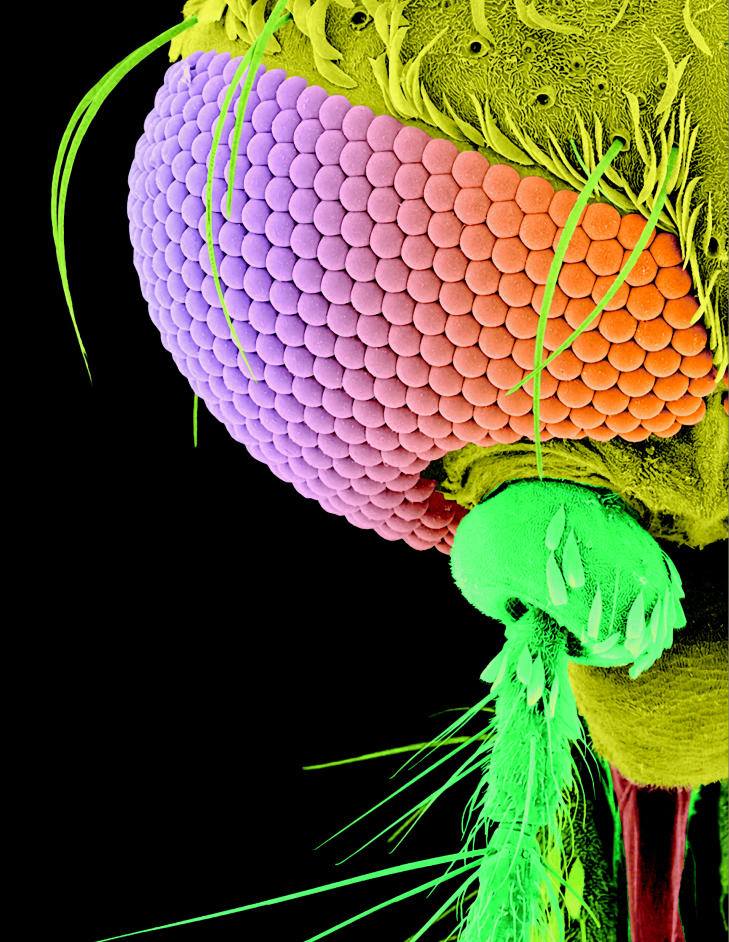


**Figure:**
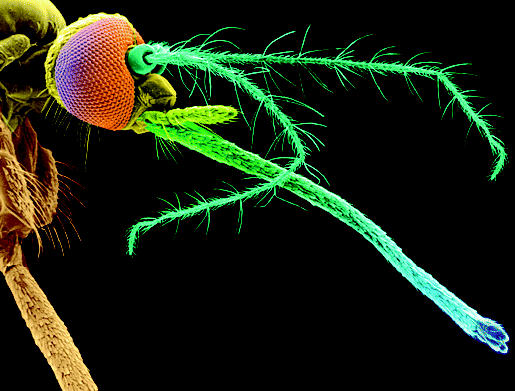
**Tuned in to scents.** Mosquitoes “smell” with their antennae.

**Figure:**
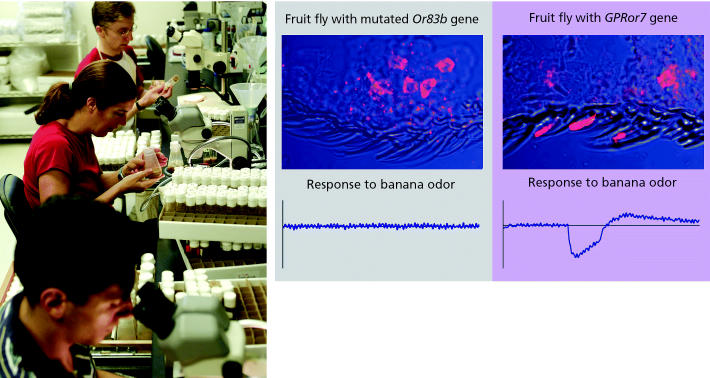
**Reception conservation.** Leslie Vosshall and colleagues (left) developed a fruit fly strain lacking the *Or83b* gene. The mutation means the fruit flies’ odorant receptors (above, stained red) are not properly localized in the sensory hairs, and the flies do not respond when exposed to banana odor. When *GPRor7*, the mosquito equivalent of *Or83b*, is put into the mutant fruit flies, the odorant receptors return to the sensory hairs, and the flies now respond to banana odor.

**Figure:**
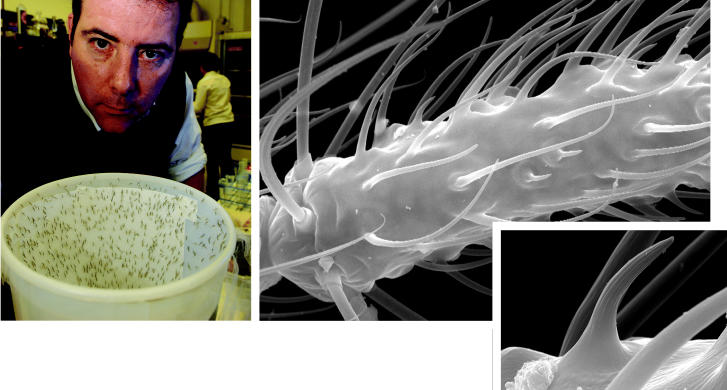
**The hair of the mosquito that bit you?** The antenna of the female *Anopheles* mosquito (above right and inset) bristles with various olfactory sensillae used for prey detection, flight direction, and egg laying. Laurence Zwiebel (above left, with mosquitoes) is targeting human-specific olfactory receptors in these mosquitoes.
